# A novel predictive score for citrate accumulation among patients receiving artificial liver support system therapy with regional citrate anticoagulation

**DOI:** 10.1038/s41598-020-69902-2

**Published:** 2020-07-30

**Authors:** Yuanji Ma, Fang Chen, Changhai Liu, Yan Xu, Ming Wang, Taoyou Zhou, Xuezhong Lei, Ping Feng, Lang Bai, Hong Tang

**Affiliations:** Center of Infectious Diseases, West China Hospital of Sichuan University, No. 37 GuoXue Xiang, Wuhou District, Chengdu, 610041 China

**Keywords:** Hepatitis, Risk factors, Continuous renal replacement therapy

## Abstract

Patients with liver failure may suffer citrate accumulation when using regional citrate anticoagulation for artificial liver support system therapy (RCA-ALSS therapy). This study aimed to develop a predictive scoring system to stratify the risk of citrate accumulation. A total of 338 patients treated with RCA-ALSS therapy were retrospectively enrolled and randomly divided into derivation and validation cohorts. Longer duration of citrate accumulation (LDCA) was defined as the presence of citrate accumulation 2 h after RCA-ALSS therapy. Four baseline variables were found to be independently associated with LDCA: gender, international normalized ratio of prothrombin time, serum creatinine, and serum chloride. A predictive R-CA model and its simplified R-CA score were developed. The R-CA model (AUROC = 0.848) was found to be superior to the MELD score (AUROC = 0.725; *p* = 0.022) and other univariate predictors (AUROCs < 0.700; all *p* ≤ 0.001) in predicting LDCA. The R-CA score (AUROC = 0.803) was as capable as the R-CA model (*p* = 0.369) and the MELD score (*p* = 0.174), and was superior to other univariate predictors (all *p* < 0.05) in predicting LDCA. An R-CA score of 0–2 had a negative predictive value of 90.2% for LDCA. Our R-CA score reliably predicts LDCA in patients with RCA-ALSS therapy, and it is easy to use. Patients with R-CA score of 0–2 can safely receive RCA-ALSS therapy, while others should be carefully evaluated before treatment.

**Trial registration**: Chinese Clinical Trial Registry, ChiCTR2000029179. Registered 17 January 2020, https://www.chictr.org.cn/showproj.aspx?proj=48084.

## Introduction

Acute-on-chronic liver failure (ACLF) is a progressive disease associated with rapid clinical deterioration and high mortality. Artificial liver support system (ALSS) therapy is an available treatment for patients with ACLF and is a bridge to liver transplantation^1^. However, the optimal extracorporeal anticoagulation regimen for ALSS therapy remains uncertain. Regional citrate anticoagulation (RCA) is now the preferred anticoagulation method for patients underwent continuous renal replacement therapy (CRRT)^2,3^. RCA seems also safe and feasible for patients with liver failure, and citrate accumulation is well tolerated by them^4–14^. The blood purification techniques used in these patients include a dialysis technique that can remove citrate directly. Our previous study suggests that RCA is relatively safe and effective in patients with ACLF receiving double plasma molecular adsorption system plus plasma exchange (DPMAS plus PE) therapy that does not include dialysis and filtration techniques^15^. However, transient citrate accumulation was found in all patients due to PE therapy in both groups and RCA in RCA group, and it remained much higher in RCA group than that in heparin anticoagulation group 2 h after the conclusion of the ALSS therapy (34.0% vs. 7.4%, *p* = 0.000)^15^.

Citrate accumulation is a feared and potentially lethal complication of RCA manifesting as an increased ratio of total calcium (Ca_tot_) to ionized calcium (Ca_ion_) with or without hypocalcaemia, metabolic acidosis and enlarged anion gap^8^. Early prediction may help avoid or reduce the risk of citrate accumulation. Schultheiß et al. found that standard laboratory liver function parameters showed poor predictive capabilities regarding citrate accumulation and that serum lactate ≥ 3.4 mmol/L and prothrombin time activity (PTA) ≤ 26% predicted an increase of citrate accumulation with high sensitivity (86% for both lactate and PTA) and specificity (86% for lactate, 92% for PTA)^8^. The results were obtained in critically ill patients with decompensated liver cirrhosis or acute liver failure treated with continuous venovenous hemodialysis (CVVHD), though they may also be useful for patients with ACLF treated with ALSS therapy that includes dialysis and filtration techniques. Our previous study revealed that gender and baseline lactate were independent predictors for citrate accumulation in patients with ACLF who received DPMAS plus PE therapy with heparin anticoagulation. In that study, an increase of citrate accumulation due to PE therapy was predicted by the presence of baseline levels of plasma lactate ≥ 2.65 mmol/L [sensitivity 62.5%, specificity 84.2%, area under the receiver operating curves (AUROCs) = 0.750, 95% confidence interval (CI) = 0.601–0.899]^16^. However, whether these results could be applied to patients with ACLF who receive ALSS therapy without dialysis and filtration techniques is unclear. In our retrospective study, we developed a model to predict longer duration of citrate accumulation (LDCA) in patients with hepatitis B virus (HBV) infection-related liver injury treated with DPMAS plus PE therapy with RCA.

## Results

### Patient characteristics

**Figure 1:**
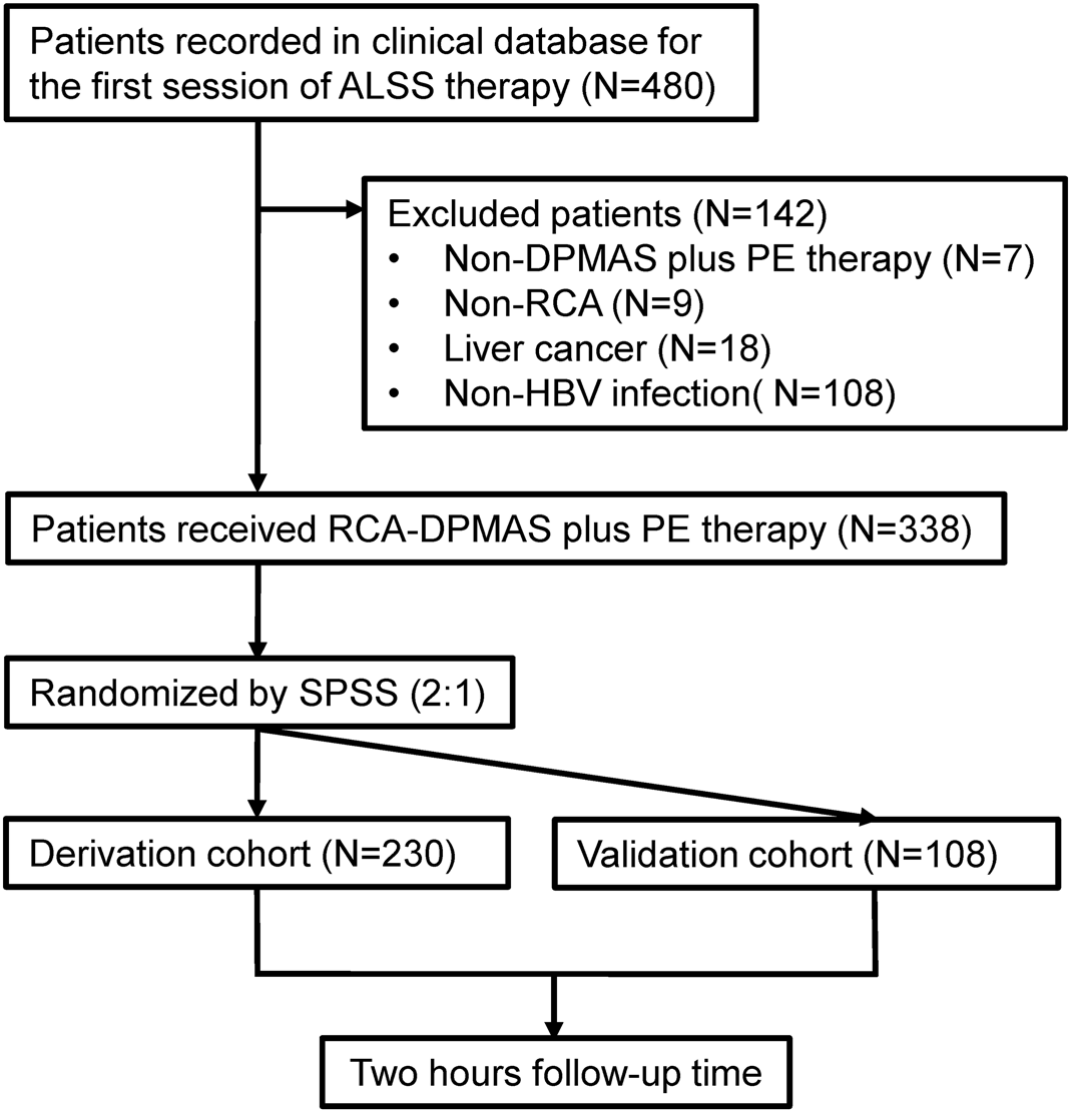
Flow diagram of patient selection. Of the 480 patients in our database that received ALSS therapy, 142 were excluded from the study. The remaining 338 patients were randomly divided into a derivation cohort (N = 230) and a validation cohort (N = 108). *ALSS* artificial liver support system, *DPMAS* double plasma molecular adsorption system, *PE* plasma exchange, *RCA* regional citrate anticoagulation, *HBV* hepatitis B virus.

A total of 480 patients treated with ALSS therapy were initially screened and enrolled (Fig. 1). Patients treated with non-DPMAS plus PE therapy (N = 7) or non-RCA (N = 9) were excluded from the study. Patients with liver cancer (N = 18) and those without HBV infection (N = 108) were also excluded. A total of 338 patients were enrolled and randomly divided into a derivation cohort (N = 230) and a validation cohort (N = 108) with a ratio of 2:1 using SPSS software. All patients were followed up 2 h after RCA-ALSS therapy.

The patient characteristics are shown in Table 1. There were no significant differences between the two cohorts in gender, age, causes of liver disease, usage of antiviral agents, or laboratory parameters before the initial ALSS therapy. The Model for End-Stage Liver Disease (MELD) score^17^ and the proportion who met the HBV-ACLF criteria^18^ were similar in the two cohorts. The overall rates of longer duration of citrate accumulation (LDCA) were not significantly different between the two cohorts. There were no significant differences in indicators representing that patients received similar RCA, such as intracorporeal Ca_tot_ before RCA-ALSS therapy, intracorporeal and extracorporeal Ca_ion_ during RCA-ALSS therapy, and intracorporeal Ca_tot_ and Ca_ion_ 2 h after RCA-ALSS therapy (Table 1).

**Table 1:** Characteristics of derivation and validation cohorts.

	Derivation cohort (N = 230)	Validation cohort (N = 108)	*p*
Female	31(13.5%)	13(12.0%)	0.713
Age (years)	45.2 ± 11.4	45.3 ± 12.4	0.953
Liver cirrhosis	160 (69.6%)	77 (71.3%)	0.746
Causes of liver disease			0.680
HBV infection only	172 (74.8%)	83 (76.9%)	
HBV infection plus other causes	58 (25.2%)	25 (23.1%)	
Antiviral therapy			0.638
Entecavir	218 (94.8%)	101 (93.5%)	
Tenofovir	12 (5.2%)	7 (6.5%)	
MELD score	25.7 ± 5.3	25.1 ± 5.1	0.293
HBV-ACLF criteria^18^	180 (78.3%)	78 (72.2%)	0.223
PT-INR	1.99 (0.88)	1.93 (0.88)	0.398
PT-INR			0.261
< 2.0	117 (50.9%)	62 (57.4%)	
≥ 2.0	113 (49.1%)	46 (42.6%)	
Serum creatinine (μmol/L)	83.5 (28.3)	85.0 (30.8)	0.652
Serum creatinine (× ULN)^#^	0.80 (0.26)	0.82 (0.30)	0.585
Serum creatinine (mg/dL)			0.357
Male: < 1.2; female: < 1.0	187 (81.3%)	85 (78.7%)	
Male: 1.2–1.5; female: 1.0–1.2	19 (8.3%)	14 (13.0%)	
Male: ≥ 1.5; female: ≥ 1.2	24 (10.4%)	9 (8.3%)	
Total bilirubin (μmol/L)	417.2 ± 122.7	405.9 ± 133.5	0.445
Direct bilirubin to total bilirubin ratio	0.80 (0.14)	0.82 (0.12)	0.347
Alanine aminotransferase (IU/L)	119 (171)	127 (209)	0.994
Aspartate aminotransferase (IU/L)	119 (117)	111 (122)	0.956
Aspartate aminotransferase to alanine aminotransferase ratio	1.03 (1.05)	1.08 (1.0)	0.477
Albumin (g/L)	32.3 ± 4.0	32.2 ± 4.4	0.995
Albumin to globulin ratio	1.23 ± 0.38	1.17 ± 0.42	0.149
Ammonia (mmol/L)	74.0 (48.3)	69.0 (44.5)	0.340
Lactate (mmol/L)	2.40 (1.05)	2.20 (1.10)	0.364
Serum sodium (mmol/L)	134.1 ± 4.5	132.7 ± 13.3	0.141
Serum potassium (mmol/L)	3.45 ± 0.57	3.39 ± 0.49	0.349
Serum chloride (mmol/L)	96.5 ± 5.2	96.4 ± 4.5	0.939
Serum chloride (mmol/L)			0.723
≥ 95	155 (67.4%)	73 (67.6%)	
90–95	50 (21.7%)	26 (24.1%)	
< 90	25 (10.9%)	9 (8.3%)	
Hemoglobin (g/L)	120 ± 20	122 ± 23	0.350
Platelets (× 10^9^/L)	102 (70)	96 (71)	0.849
White blood cells (× 10^9^/L)	7.4 ± 3.3	7.2 ± 3.7	0.570
R-CA model	-1.95 (1.72)	-1.91 (1.69)	0.661
R-CA score	2 (3)	2 (3)	0.567
Intracorporeal Ca_tot_ before RCA-ALSS therapy (mmol/L)	2.15 ± 0.13	2.14 ± 0.16	0.957
Intracorporeal Ca_ion_ during RCA-ALSS therapy (mmol/L)	0.791 ± 0.113	0.813 ± 0.112	0.197
Extracorporeal Ca_ion_ during RCA-ALSS therapy (mmol/L)	0.178 (0.094)	0.177 (0.090)	0.219
Intracorporeal Ca_tot_ 2 h after RCA-ALSS therapy (mmol/L)	2.51 ± 0.21	2.52 ± 0.22	0.685
Intracorporeal Ca_ion_ 2 h after RCA-ALSS therapy (mmol/L)	1.069 ± 0.108	1.072 ± 0.115	0.795
Ca_tot_/Ca_ion_ 2 h after RCA-ALSS therapy	2.28 (0.33)	2.27 (0.44)	0.758
LDCA	54 (23.5%)	34 (31.5%)	0.118

Measurement data are represented as mean ± SD (normally distributed data) or median (IQR) (non-normally distributed data). Enumeration data are represented as frequencies (proportion).

*MELD* Model for End-Stage Liver Disease, *HBV* hepatitis B virus, *ACLF* acute-on-chronic liver failure, *PT-INR* international normalized ratio (INR) of prothrombin time (PT), *ULN* upper limit of normal, *RCA-ALSS therapy* artificial liver support system therapy with regional citrate anticoagulation, *Ca*_*tot*_ total calcium, *Ca*_*ion*_ ionized calcium, *Ca*_*tot*_*/Ca*_*ion*_ Ca_tot_ to Ca_ion_ ratio, *R-CA model* logistic regression model of risk predictors for citrate accumulation, *R-CA score* risk score for citrate accumulation, *LDCA* longer duration of citrate accumulation, *RCA* regional citrate anticoagulation, *ALSS therapy* artificial liver support system therapy.

^#^Statistical analysis using relative values adjusted by gender (a multiple of upper limit of normal).

### Development of R-CA model in derivation cohort

The derivation cohort was analyzed for the predictors of LDCA on the basis of baseline parameters by logistic regression analysis. In multivariate analysis, four baseline variables were found to be independently associated with LDCA: gender, international normalized ratio (INR) of prothrombin time (PT) (PT-INR), serum creatinine, and serum chloride (Table 2). A predictive R-CA model, Logit (*P*) = 5.380 + 1.173 × Gender + 0.797 × PT-INR + 1.863 × Serum creatinine (× ULN)^#^ − 0.109 × Serum chloride (mmol/L) [gender: female = 2, male = 0; ^#^Statistical analysis using relative values adjusted by gender (a multiple of upper limit of normal)], was developed by using multivariate logistic regression analysis with the backward stepwise (likelihood ratio) method.

**Table 2:** Predictors for LDCA in derivation cohort.

Predictor	Univariate			Multivariate		
	HR	95% CI	*p*	HR	95% CI	*p*
Female	7.42	3.30–16.67	0.000	10.45	4.06–26.89	0.000
Age (years)	1.06	1.03–1.09	0.000			
Liver cirrhosis	1.97	0.95–4.10	0.069			
PT-INR	2.43	1.50–3.93	0.000	2.22	1.25–3.94	0.007
Total bilirubin (μmol/L)	1.00	1.00–1.00	0.492			
Albumin (g/L)	0.96	0.89–1.04	0.316			
Serum creatinine (× ULN)^#^	5.91	2.45–14.28	0.000	6.44	2.40–17.34	0.000
Serum sodium (mmol/L)	0.91	0.85–0.98	0.007			
Serum potassium (mmol/L)	0.83	0.48–1.44	0.503			
Serum chloride (mmol/L)	0.89	0.84–0.94	0.000	0.90	0.84–0.96	0.002
Lactate (mmol/L)	1.22	1.01–1.48	0.036			
Ammonia (mmol/L)	1.00	0.99–1.01	0.777			
Hemoglobin (g/L)	0.98	0.96–0.99	0.002			
Platelets (× 10^9^/L)	1.00	0.99–1.00	0.143			
White blood cells (× 10^9^/L)	1.05	0.97–1.15	0.245			

*LDCA* longer duration of citrate accumulation, *HR* hazard ratio, *CI* confidence interval, *PT-INR* international normalized ratio (INR) of prothrombin time (PT), *ULN* upper limit of normal.

^#^Statistical analysis using relative values adjusted by gender (a multiple of upper limit of normal).

### Testing of R-CA model in validation cohort

**Figure 2:**
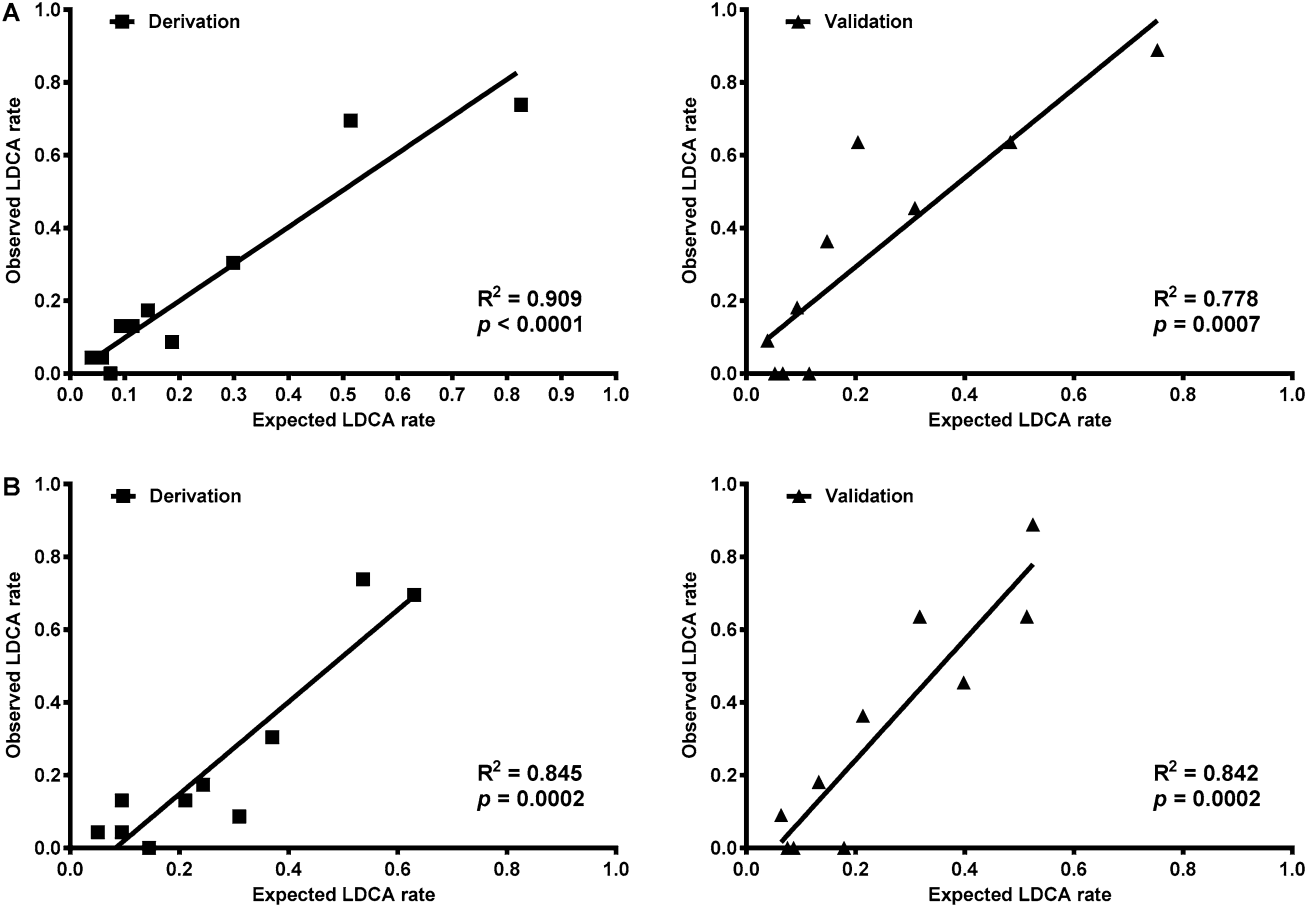
Linear correlation lines of expected LDCA rate and observed LDCA rate of R-CA model and R-CA score. (**A**) The linear correlation lines of expected and observed LDCA rates in the derivation and validation cohorts based on the R-CA model. (**B**) The linear correlation lines of expected and observed LDCA rates in the derivation and validation cohorts based on the R-CA score. The expected and observed LDCA rates of the derivation cohort match those of the validation cohort. *LDCA* longer duration of citrate accumulation, *R-CA model* logistic regression model of risk predictors for citrate accumulation, *R-CA score* risk score for citrate accumulation.

Before developing a simplified predictive score, the four independent predictors were tested in the validation cohort. Gender, PT-INR, serum creatinine, and serum chloride were verified as independent predictors of LDCA based on the results of the multivariate analysis of the validation cohort (Table 3). The R-CA model showed a good predictability with AUROC of 0.848 (95% CI 0.795–0.892, *p* = 0.000) and 0.856 (95% CI 0.776–0.916, *p* = 0.000) in the derivation and validation cohorts, respectively. The expected LDCA rates and observed LDCA rates from the derivation cohort (R^2^ = 0.909, *p* = 0.000) matched with the validation cohort (R^2^ = 0.778, *p* = 0.007; Fig. 2A).

**Table 3:** Testing predictors for LDCA in validation cohort.

Predictor	Multivariate		
	HR	95% CI	*p*
Female	5.98	1.37–26.06	0.017
PT-INR	4.07	1.62–10.25	0.003
Serum creatinine (× ULN)^#^	78.02	5.71–1,064.90	0.001
Serum chloride (mmol/L)	0.86	0.77–0.97	0.015

*LDCA* longer duration of citrate accumulation, *HR* hazard ratio, *CI* confidence interval, *PT-INR* international normalized ratio (INR) of prothrombin time (PT), *ULN* upper limit of normal.

^#^Statistical analysis using relative values adjusted by gender (a multiple of upper limit of normal).

### Development of R-CA score

Following the validation of our predictive model, the four individual parameters were scored. An ordinal grading system (0–2) with distinct hazard ratios (HR) on logistic regression was performed by comprehensively considering their cut-off values of AUROCs predicting the probability of LDCA, ACLF diagnostic criteria^19,20^, and clinically significant values (Tables 4, 5). The total R-CA score ranges from a minimum of 0 to a maximum of 8. The scoring parameters are easy to collect laboratory measurements or clinical features that showed a distinct hazard ratio on logistic regression in derivation and validation cohorts (all HR > 2 and all *p* = 0.000; Table 6).

**Table 4:** Simplified univariate predictors for LDCA in derivation and validation cohorts.

Predictor	Derivation cohort			Validation cohort		
	HR	95% CI	*p*	HR	95% CI	*p*
Gender						
Male	1	–	–	1	–	–
Female	7.42	3.30–16.67	0.000	4.25	1.27–14.17	0.019
PT-INR						
< 2.0	1	–	–	1	–	–
≥ 2.0	2.57	1.36–4.87	0.004	2.64	1.15–6.07	0.023
Serum creatinine (mg/dL)						
Male: < 1.2; Female: < 1.0	1	–	–	1	–	–
Male: 1.2–1.5; Female: 1.0–1.2	3.05	1.11–8.39	0.030	4.63	1.43–15.00	0.011
Male: ≥ 1.5; Female: ≥ 1.2	12.71	4.85–33.29	0.000	12.16	2.33–63.45	0.003
Serum chloride (mmol/L)						
≥ 95	1	–	–	1	–	–
90–95	2.68	1.30–5.53	0.008	3.05	1.18–7.89	0.021
< 90	4.80	1.96–11.73	0.001	7.13	1.60–31.70	0.010

*LDCA* longer duration of citrate accumulation, *HR* hazard ratio, *CI* confidence interval, *PT-INR* international normalized ratio (INR) of prothrombin time (PT).

**Table 5:** R-CA score.

Points	Gender	PT-INR	Serum creatinine (mg/dL)		Serum chloride (mmol/L)
			Male	Female	
0	Male	< 2.0	< 1.2	< 1.0	≥ 95
1			1.2–1.5	1.0–1.2	90–95
2	Female	≥ 2.0	≥ 1.5	≥ 1.2	< 90

*R-CA score* risk score for citrate accumulation, *PT-INR* international normalized ratio (INR) of prothrombin time (PT).

**Table 6:** Predictive model for LDCA in derivation and validation cohorts, and that among patients with or without liver cirrhosis.

Subject	R-CA model			R-CA score		
	HR	95% CI	*p*	HR	95% CI	*p*
Derivation cohort	2.72	2.02–3.65	0.000	2.16	1.71–2.72	0.000
Validation cohort	3.53	2.13–5.84	0.000	2.51	1.72–3.65	0.000
Patients with liver cirrhosis	3.14	2.29–4.30	0.000	2.25	1.77–2.86	0.000
Patients without liver cirrhosis	2.31	1.52–3.51	0.000	2.11	1.47–3.02	0.000

*LDCA* longer duration of citrate accumulation, *R-CA model* logistic regression model of risk predictors for citrate accumulation, *R-CA score* risk score for citrate accumulation, *HR* hazard ratio, *CI* confidence interval, *PT-INR* international normalized ratio (INR) of prothrombin time (PT).

**Figure 3:**
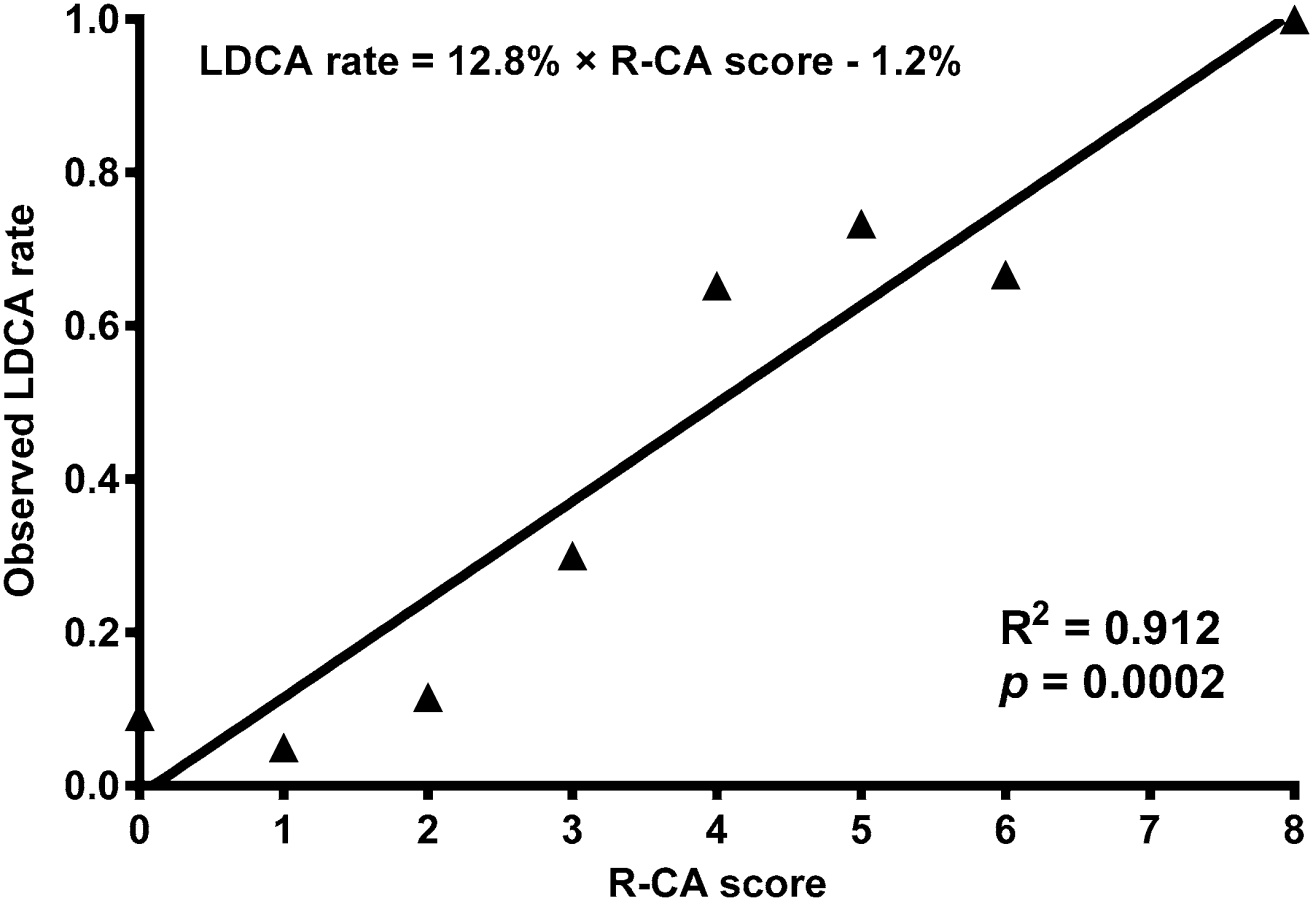
Linear regression lines of R-CA score and observed LDCA rate in the derivation cohort. A linear regression equation was developed for the R-CA scores and LDCA in derivation. *LDCA* longer duration of citrate accumulation, *R-CA score* risk score for citrate accumulation.

The R-CA scores and LDCA rates showed a linear correlation in the derivation cohort (R^2^ = 0.912, *p* = 0.000; Fig. 3). A linear regression equation was developed: LDCA rate = 12.8% × R-CA score − 1.2%. The expected LDCA rates and observed LDCA rates based on the R-CA scores in the derivation cohort (R^2^ = 0.845, *p* = 0.000) matched those of the validation cohort (R^2^ = 0.842, *p* = 0.000; Fig. 2B).

### Evaluation of R-CA model and R-CA score as predictors of LDCA in derivation and validation cohorts

**Figure 4:**
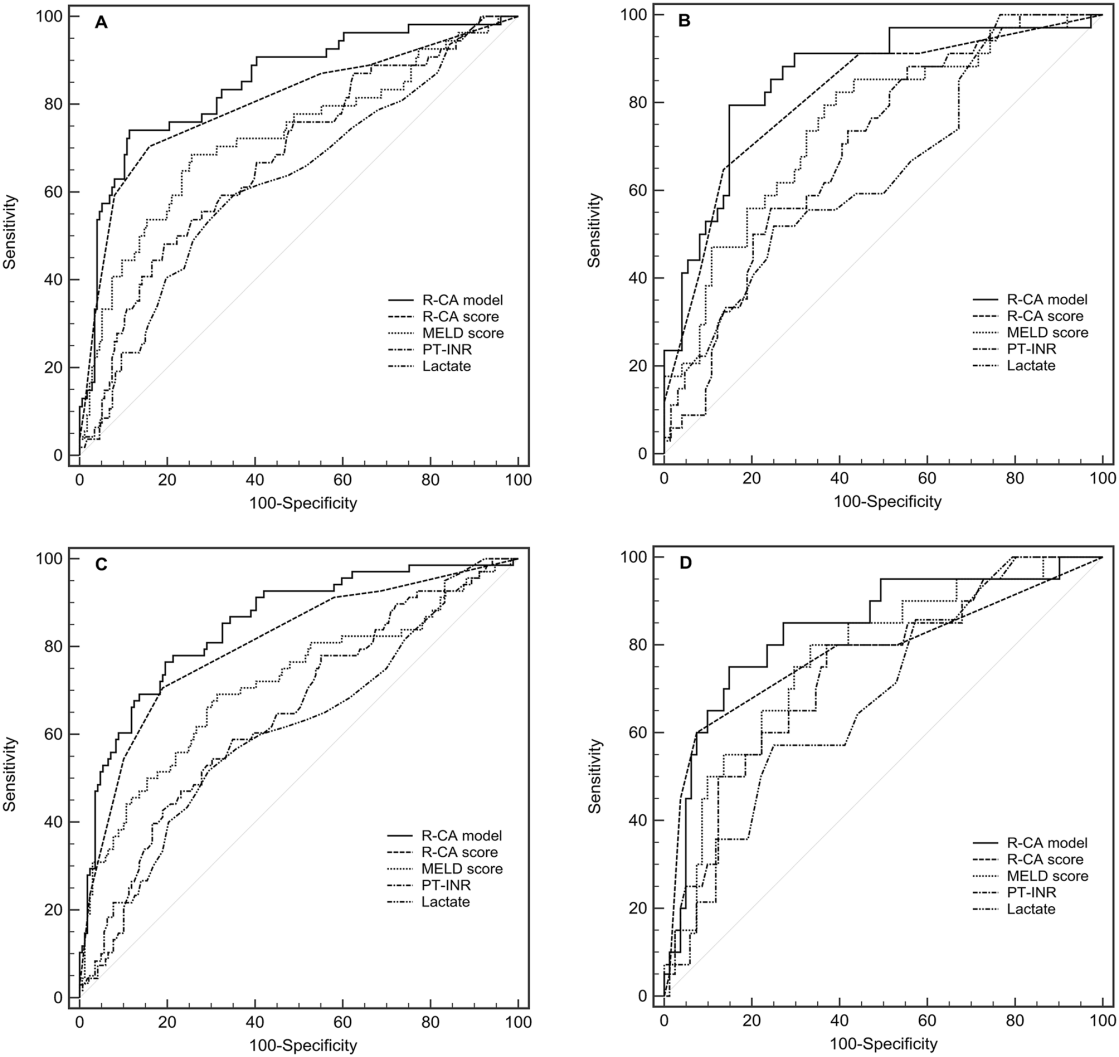
Receiver operating curves (ROC) for the abilities of risk models to predict LDCA. ROC for risk models predicting 3-month mortality in the derivation cohort (**A**), validation cohort (**B**), patients with liver cirrhosis (**C**), and patients without liver cirrhosis (**D**). Our R-CA model and R-CA score were as capable as or superior to all other models in predicting LDCA. *R-CA model* logistic regression model of risk predictors for citrate accumulation, *R-CA score* risk score for citrate accumulation, *MELD* Model for End-Stage Liver Disease, *PT-INR* international normalized ratio (INR) of prothrombin time (PT).

Our R-CA model and its simplified R-CA score were compared with other potential predictors, such as the MELD score, PT-INR and lactate (Fig. 4A,B and Table 7). AUROCs of the R-CA model and the R-CA score in the derivation cohort were 0.848 and 0.803, and those in the validation cohort were 0.856 and 0.816, respectively. Our R-CA model was found to be as capable as the R-CA score, and superior to the MELD score (AUROC = 0.725) and other univariate predictors (AUROCs < 0.700), in predicting LDCA (*p* = 0.369, *p* = 0.022 and *p* ≤ 0.001, respectively). The R-CA score was as capable as the MELD score in predicting LDCA (*p* = 0.174) and superior to other univariate predictors in predicting LDCA (*p* < 0.05).

**Table 7:** Comparison of the predictive values of R-CA model, R-CA score, and other predictors in derivation and validation cohorts.

Predictor	Derivation cohort				Validation cohort			
	AUROC	95% CI	*Z*	*p*	AUROC	95% CI	*Z*	*p*
R-CA model	0.848	0.795–0.892			0.856	0.776–0.916		
R-CA score	0.803	0.746–0.853	0.90	0.369	0.816	0.730–0.884	0.67	0.503
MELD score	0.725	0.662–0.781	2.29	0.022	0.751	0.659–0.829	1.61	0.107
PT-INR	0.678	0.613–0.738	3.22	0.001	0.697	0.601–0.781	2.43	0.015
Lactate	0.627	0.554–0.695	3.85	0.000	0.647	0.540–0.744	2.79	0.005
R-CA score	0.803	0.746–0.853			0.816	0.730–0.884		
MELD score	0.725	0.662–0.781	1.36	0.174	0.751	0.659–0.829	0.96	0.336
PT-INR	0.678	0.613–0.738	2.21	0.027	0.697	0.601–0.781	1.75	0.080
Lactate	0.627	0.554–0.695	2.89	0.004	0.647	0.540–0.744	2.19	0.028

Area under the ROC curves (AUROCs) for different models were calculated and compared using the Z test (Delong's method).

*R-CA model* logistic regression model of risk predictors for citrate accumulation, *R-CA score* risk score for citrate accumulation, *CI* confidence interval, *MELD* Model for End-Stage Liver Disease, *PT-INR* international normalized ratio (INR) of prothrombin time (PT).

Less than 10% of patients who had an R-CA model ≤  − 1.00 or an R-CA score of 0–2 experienced LDCA. R-CA model ≤  − 1.00 had an AUROC of 0.848, a sensitivity of 74.1%, a specificity of 88.6%, a positive predictive value of 66.7%, and a negative predictive value of 91.8%. R-CA score of 0–2 had an AUROC of 0.803, a sensitivity of 70.4%, a specificity of 84.1%, a positive predictive value of 57.6%, and a negative predictive value of 90.2% (Table 8). Although patients with MELD score ≤ 26.6, PT-INR ≤ 2.43 and lactate ≤ 2.65 had high negative predictive values of LDCA, their AUROCs were all less than 0.750 (Table 8).

**Table 8:** Predictive values of predictors based on their maximum area of AUROCs in derivation cohort and patients with liver cirrhosis.

Subject	Predictor	AUROC	Cut-off	Sensitivity (%)	Specificity (%)	Positive predictive value (%)	Negative predictive value (%)
Derivation	R-CA model	0.848	− 1.00	74.1	88.6	66.7	91.8
	R-CA score	0.803	2.5^#^	70.4	84.1	57.6	90.2
	MELD score	0.725	26.6	68.5	74.4	45.1	88.5
	PT-INR	0.678	2.43	48.1	80.7	43.3	83.5
	Lactate	0.627	2.65	51.9	75.0	36.8	82.1
Patients with liver cirrhosis	R-CA model	0.851	− 1.39	76.5	80.5	61.2	89.5
	R-CA score	0.804	2.5^#^	70.6	81.1	60.0	87.3
	MELD score	0.712	26.6	69.1	68.6	47.0	84.7
	PT-INR	0.646	2.22	54.4	69.8	42.0	79.2
	Lactate	0.613	2.65	51.7	70.6	42.5	77.4

*AUROC* area under the ROC curves, *R-CA model* logistic regression model of risk predictors for citrate accumulation, *R-CA score* risk score for citrate accumulation, *MELD* Model for End-Stage Liver Disease, *PT-INR* international normalized ratio (INR) of prothrombin time (PT).

^#^No decimal allowed.

### Evaluation of R-CA model and R-CA score as predictors of LDCA among patients with or without liver cirrhosis

**Figure 5:**
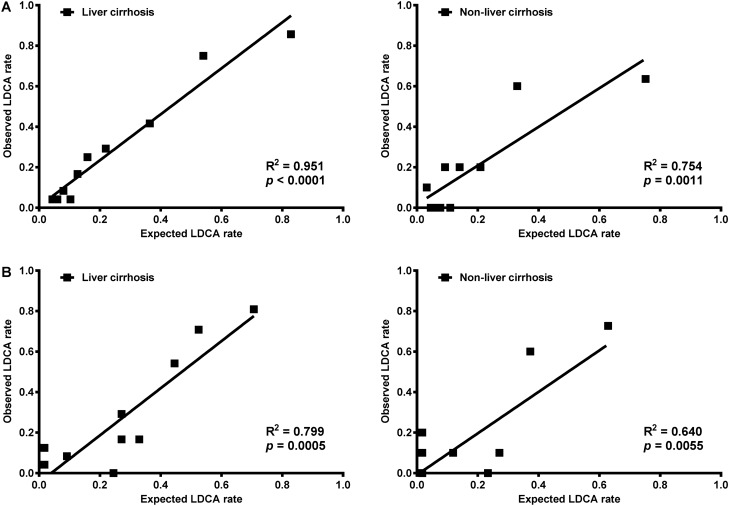
Linear correlation lines of expected LDCA rate and observed LDCA rate of R-CA model and R-CA score among patients with or without liver cirrhosis. (**A**) The linear correlation lines of expected and observed LDCA rates among patients with or without liver cirrhosis based on the R-CA model. (**B**) The linear correlation lines of expected and observed LDCA rates among patients with or without liver cirrhosis based on the R-CA score. The expected and observed LDCA rates of patients with liver cirrhosis match those of patients without liver cirrhosis. *LDCA* longer duration of citrate accumulation, *R-CA model* logistic regression model of risk predictors for citrate accumulation, *R-CA score* risk score for citrate accumulation.

The R-CA model and R-CA score also showed a distinct hazard ratio on logistic regression among patients with or without liver cirrhosis (all HR > 2 and all *p* = 0.000; Table 6). The expected LDCA rates and observed LDCA rates based on the R-CA model among patients with liver cirrhosis (R^2^ = 0.951, *p* = 0.000) matched with patients without liver cirrhosis (R^2^ = 0.754, *p* = 0.001; Fig. 5A). The expected LDCA rates and observed LDCA rates based on the R-CA scores among patients with liver cirrhosis (R^2^ = 0.799, *p* = 0.001) also matched those of patients without liver cirrhosis (R^2^ = 0.640, *p* = 0.006; Fig. 5B).

AUROCs of the R-CA model and the R-CA score among patients with liver cirrhosis were 0.851 and 0.804, and those among patients without liver cirrhosis were 0.836 and 0.781, respectively (Fig. 4C,D, and Table 9). Our R-CA model was found to be as capable as the R-CA score, and superior to the MELD score (AUROC = 0.712) and other univariate predictors (AUROCs < 0.700), in predicting LDCA (*p* = 0.280, *p* = 0.005 and *p* = 0.000, respectively). The R-CA score was as capable as the MELD score in predicting LDCA (*p* = 0.075) and superior to other univariate predictors in predicting LDCA (*p* < 0.01).

**Table 9:** Comparison of the predictive values of R-CA model, R-CA score, and other predictors among patients with or without liver cirrhosis.

Predictor	Patients with liver cirrhosis				Patients without liver cirrhosis			
	AUROC	95% CI	*Z*	*p*	AUROC	95% CI	*Z*	*p*
R-CA model	0.851	0.799–0.893			0.836	0.749–0.902		
R-CA score	0.804	0.748–0.853	1.08	0.280	0.781	0.688–0.858	0.62	0.533
MELD score	0.712	0.650–0.769	2.81	0.005	0.770	0.675–0.848	0.82	0.412
PT-INR	0.646	0.582–0.707	4.22	0.000	0.739	0.642–0.822	1.17	0.242
Lactate	0.613	0.542–0.680	4.53	0.000	0.680	0.568–0.779	1.668	0.095
R-CA score	0.804	0.748–0.853			0.781	0.688–0.858		
MELD score	0.712	0.650–0.769	1.78	0.075	0.770	0.675–0.848	0.13	0.896
PT-INR	0.646	0.582–0.707	3.11	0.002	0.739	0.642–0.822	0.46	0.645
Lactate	0.613	0.542–0.680	3.50	0.001	0.680	0.568–0.779	1.00	0.318

Area under the ROC curves (AUROCs) for different models were calculated and compared using the Z test (Delong's method).

*R-CA model* logistic regression model of risk predictors for citrate accumulation, *R-CA score* risk score for citrate accumulation, *CI* confidence interval, *MELD* Model for End-Stage Liver Disease, *PT-INR* international normalized ratio (INR) of prothrombin time (PT).

About 12.5% of cirrhotic patients who had an R-CA model ≤  − 1.39 or an R-CA score of 0–2 experienced LDCA. R-CA model ≤  − 1.39 had an AUROC of 0.851, a sensitivity of 76.5%, a specificity of 80.5%, a positive predictive value of 61.2%, and a negative predictive value of 89.5%. R-CA score of 0–2 had an AUROC of 0.804, a sensitivity of 70.6%, a specificity of 81.1%, a positive predictive value of 60.0%, and a negative predictive value of 87.3% (Table 8). Although cirrhotic patients with MELD score ≤ 26.6, PT-INR ≤ 2.22 and lactate ≤ 2.65 had high negative predictive values of LDCA, their AUROCs were all less than 0.750 (Table 8).

### Correlation between R-CA model, R-CA score, LDCA and disease severity

**Figure 6:**
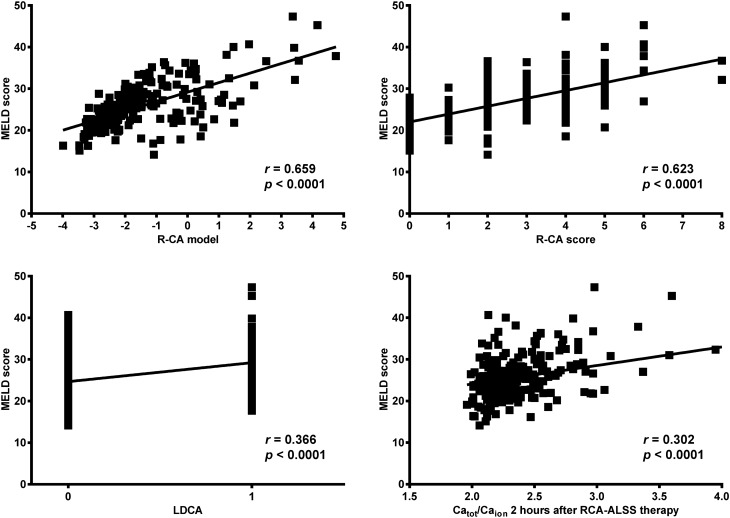
Correlation between R-CA model, R-CA score, LDCA and disease severity in derivation cohort. R-CA model, R-CA score and LDCA are positively correlated with disease severity rated by MELD score. *MELD* Model for End-Stage Liver Disease, *R-CA model* logistic regression model of risk predictors for citrate accumulation, *R-CA score* risk score for citrate accumulation, *LDCA* longer duration of citrate accumulation, *Ca*_*tot*_ total calcium, *Ca*_*ion*_ ionized calcium, *Ca*_*tot*_*/Ca*_*ion*_ Ca_tot_ to Ca_ion_ ratio, *RCA* regional citrate anticoagulation, *ALSS therapy* artificial liver support system therapy.

R-CA model, R-CA score, LDCA, and Ca_tot_/Ca_ion_ 2 h after RCA-ALSS therapy were positively correlated with disease severity rated by MELD score in derivation and validation cohorts and among patients with or without liver cirrhosis with all the *p* < 0.01 (Fig. 6, Table 10).

**Table 10:** Correlation of R-CA model, R-CA score, LDCA and disease severity rated by MELD score in derivation and validation cohorts, and that among patients with or without liver cirrhosis.

Subject	R-CA model		R-CA score		LDCA		Ca_tot_/Ca_ion_ 2 h after therapy	
	*r*	*p*	*r*	*p*	*r*	*p*	*r*	*p*
Derivation cohort	0.659	0.000	0.623	0.000	0.302	0.000	0.366	0.000
Validation cohort	0.600	0.000	0.585	0.000	0.488	0.000	0.426	0.000
Patients with liver cirrhosis	0.625	0.000	0.607	0.000	0.390	0.000	0.367	0.000
Patients without liver cirrhosis	0.660	0.000	0.585	0.000	0.289	0.003	0.379	0.000

*R-CA model* logistic regression model of risk predictors for citrate accumulation, *R-CA score* risk score for citrate accumulation, *MELD* Model for End-Stage Liver Disease, *Ca*_*tot*_ total calcium, *Ca*_*ion*_ ionized calcium, *Ca*_*tot*_*/Ca*_*ion*_ Ca_tot_ to Ca_ion_ ratio.

## Discussion

We developed a predictive model of citrate accumulation for patients with liver failure treated with RCA-ALSS therapy. We found that four variables (gender, PT-INR, serum creatinine, and serum chloride) are independent predictors of LDCA. A predictive R-CA model and its simplified R-CA score have both been developed. Prior to our study, the existing predictors in the literature were all univariate, such as PT-INR and lactate^8,16^. There was no score dedicated to LDCA in patients with liver failure treated with RCA-ALSS therapy before our study.

Our R-CA model was constructed based on the four most significant variables found in our multivariate analysis. Female gender, serum creatinine, and PT-INR were positively correlated with LDCA, while serum chloride was negatively correlated. It has been reported that the metabolism of citrate is mainly in the mitochondria of liver, muscle, and kidney cells^21^. This helps to explain the positive correlations between LDCA and gender, serum creatinine, and PT-INR. LDCA may be more likely to occur in female patients because, on average, men have more muscle mass than women, allowing for more citrate metabolism in muscle cells^22^. As serum creatinine is a marker for renal function, it is expected that both citrate and serum creatinine levels will be high if metabolism in the kidneys is slowed. Additionally, as coagulation failure is a symptom of ACLF, and PT-INR values have been correlated with the degree of liver injury, which is related to the function of liver cells’ mitochondria, a high PT-INR indicates a lower ability of liver cells to metabolize citrate^1^. The negative correlation between serum chloride and LDCA may be explained by the anion gap. Chloride is one of the most important anions in the body, and its decrease may indicate the increase of other anions, such as lactate, to maintain the anion gap. The correlation coefficient between serum chloride and lactate is − 0.173 (*p* = 0.001) (Data not shown). Our previous study reveals that baseline Ca_tot_/Ca_ion_ is an independent predictor for citrate accumulation in patients with ACLF who received DPMAS plus PE therapy with heparin anticoagulation (AUROC = 0.725)^16^. However, the role of the anion gap in predicting LDCA requires further investigation.

We found no correlation between LDCA and standard laboratory liver function parameters, which is in line with the results of previous studies^9,12^. We predicted that age may be negatively correlated with LDCA, due to decreased muscle mass and weakened mitochondria^23,24^, but that is not supported by our study.

Our R-CA model is based on some of the same parameters used to determine the MELD score (serum creatinine and PT-INR), which has been used for organ allocation in patients with end-stage liver disease^17^. We found an AUROC of 0.725 when using the MELD score to predict LDCA, which is inferior to our R-CA model. Our results did not support lactate and PT-INR as predictive factors, though they have been reported in previous studies^8,16^. Although our R-CA model is able to successfully predict LDCA, complex calculations are required, much like when calculating a MELD score^17^. To make the model more useful, we developed the R-CA score, a simplified version that does not require complex variables and can be used easily at the patient’s bedside. The R-CA score is constructed based on the four most significant and readily available variables found in our multivariate analysis, and it is as capable as our R-CA model and the MELD score in predicting LDCA. Thus, it has a distinct advantage over our R-CA model and the MELD score. The simple scoring system assigns 0–2 points for each of the four parameters, much like the Child–Turcotte–Pugh (CTP) score for patients with liver cirrhosis and the CLIF-C ACLF score for patients with liver failure^25,26^. The cutoff values were determined using AUROC, ACLF diagnostic criteria^19,20^, and clinically significant values. The R-CA score was verified using a validation cohort, and found to be successful in predicting LDCA (AUROC = 0.803 and 0.816 in the derivation and validation cohorts, respectively). The CLIF-C ACLF score, one of the most important but easy-to-use prognostic scores for disease severity in patients with ACLF^26^, probably also has the capability in predicting LDCA because of some of the same parameters (serum creatinine and PT-INR). We had tried to compare the CLIF-C ACLF score to our score, but the respiratory indicator (arterial blood gas analysis result) was missing in most cases in this retrospectively study. The role of CLIF-C ACLF score in predicting LDCA requires further investigation.

In critically ill patients with acute kidney injury without liver disease treated with RCA-CVVHD, ROC data supports lactate (cutoff value 2.39 mmol/L) as a strong negative predictive factor of citrate accumulation, with a predictive value of 99.28%^27^. In our study, lactate has a negative predictive value of 82.1% at a cutoff of 2.65 mmol/L, but its AUROC is only 0.627, and it was not found to be an independent risk factor. These results agree with those of our previous study^16^. Similarly, the MELD score and PT-INR also showed high negative predictive values but had low AUROCs (AUROCs < 0.750).

In our study, an R-CA score 0–2 predicted the absence of LDCA with a negative predictive value of 90.2%. Approximately 70% of the patients in this study had an R-CA score 0–2. In addition to predicting LDCA, the R-CA score may have the potential to guide initial anticoagulant prescriptions in the future. Patients with an R-CA score 0–2 can safely receive RCA, but the initial dosage should be reduced, and intervention may be needed, in patients with an R-CA score of 3–8. Automated anticoagulant technology may provide a user-friendly and safe system for patients with liver failure treated with RCA-ALSS therapy in the future^28^.

In this study, we found that R-CA model, R-CA score, and LDCA were positively correlated with disease severity rated by MELD score. As the R-CA model and R-CA score are based on some of the same parameters as the MELD score^17^, the CLIF-C ACLF score^26^, and the ACLF criteria^19,20^, and an elevated MELD score, CLIF-C ACLF score, and Ca_tot_/Ca_ion_ being associated with increased mortality in patients with liver failure^17,26,29^, the R-CA model, R-CA score, and LDCA could also have the ability to predict patients’ prognosis. The direct relationship between these values and patients’ prognosis requires further investigation.

Our study has several limitations. First, as a retrospective study with a small number of patients from a single geographic area, the patient characteristics may not represent the general population, which may have affected the results of our study. Second, the patients in our study all had chronic HBV infections presenting as acute hepatic decompensation. Data derived from this subset may not be applicable to all patients with liver disease, and requires further investigation. Lastly, we used the Ca_tot_/Ca_ion_ ratio instead of a direct measurement of plasma citrate concentration to reflect citrate accumulation. The upper normal and toxic levels of citrate in the blood are not well established, and as citrate is a physiological metabolite, it may not be toxic, but instead lead to metabolic lag when it accumulates^8,15^. Further investigation on normal citrate levels, as well as the effect of citrate accumulation in the plasma, is needed.

## Conclusions

Our R-CA model, based on gender, PT-INR, serum creatinine and serum chloride, reliably predicts LDCA in patients with HBV-ACLF who are undergoing RCA-ALSS therapy. The simplified R-CA score is also reliable, and easy to use. Patients with an R-CA score 0–2 can safely receive RCA-ALSS therapy, while others should be carefully evaluated and monitored during treatment. Our model requires further validation via prospective cohort studies.

## Methods

### Study design and patients

Patients treated with ALSS therapy were recorded in a previously established clinical database from the Center of Infectious Diseases, West China Hospital of Sichuan University since January 2014. All ALSS therapies were evaluated on a case-by-case basis by treating physician referring to the Guideline for Diagnosis and Treatment of Liver Failure drawn up by Chinese Medical Association^30^. Three main indications for ALSS therapy, liver failure or pre-liver failure, severe hyperbilirubinemia with no response to medicine, perioperative period of liver transplantation for end-stage liver disease, are recommended. RCA would be implemented if there were no contraindications (circulatory shock, or hypoxemia that cannot be corrected by oxygen therapy) other than abnormal liver function since January 2018. Patients treated with ALSS therapy between January 2018 and December 2019 were retrospectively included in this study (N = 480; Fig. 1). Patients treated with non-DPMAS plus PE therapy (N = 7) or non-RCA (N = 9) were excluded from the study. Patients with liver cancer (N = 18) and those without HBV infection (N = 108) were also excluded. The remaining 338 patients were randomly divided into two cohorts: a derivation cohort (N = 230), used to develop our R-CA model; and a validation cohort (N = 108), used to test our R-CA model. All patients were followed up 2 h after RCA-ALSS therapy. Approval for this study was obtained from the Biomedical Research Ethics Committee of West China Hospital of Sichuan University. All study components were performed according to the ethical standards laid down in the 1964 Declaration of Helsinki and its later amendments. Informed consent was obtained from all subjects or, if subjects were under 18, from a parent and/or legal guardian.

### RCA-ALSS therapy

**Figure 7:**
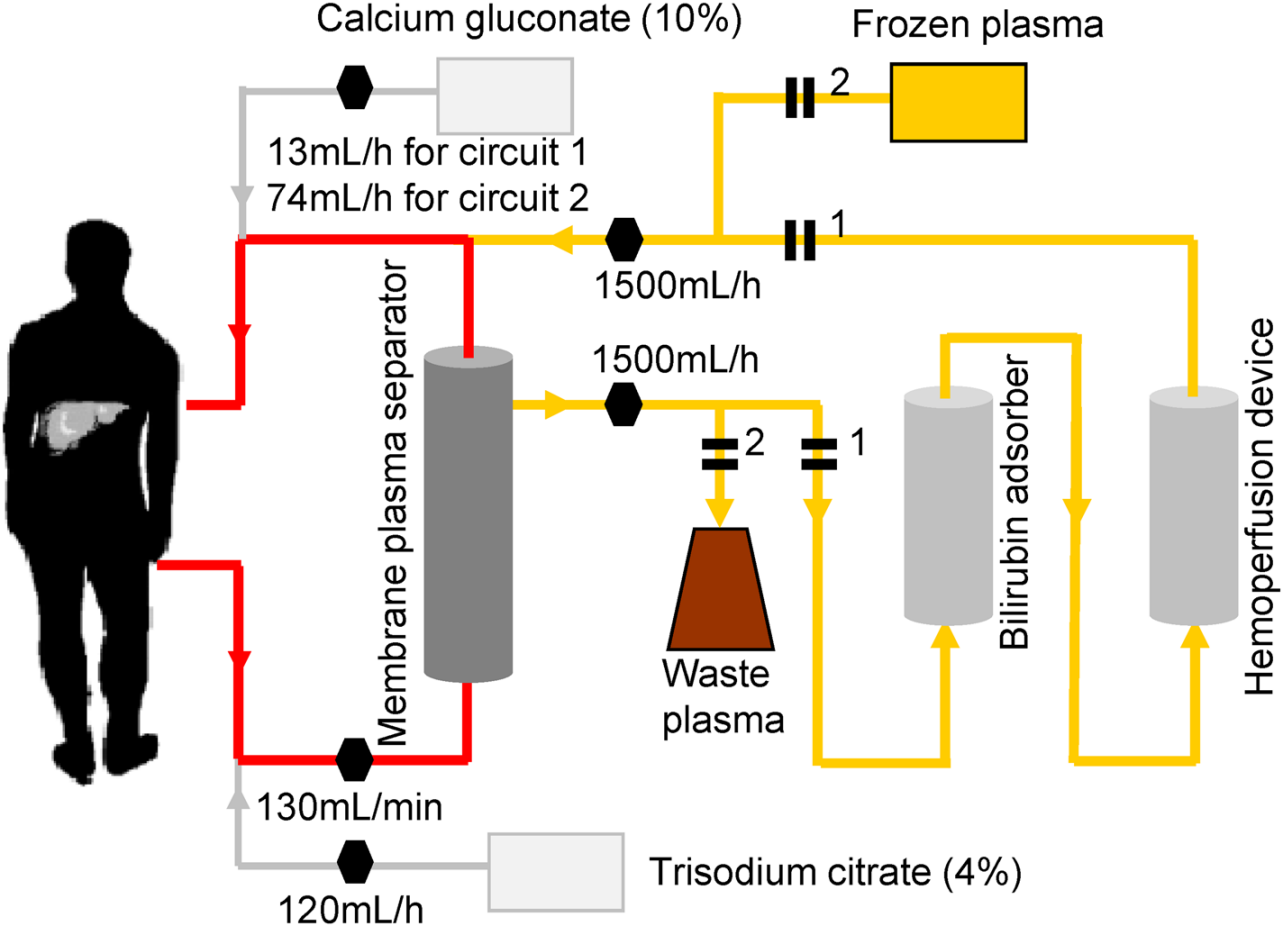
Schematic diagram of the artificial liver support system therapy with regional citrate anticoagulation. Circuit 2 therapy is initiated immediately when circuit 1 therapy is completed.

All patients received standard medicinal treatment and DPMAS plus PE therapy. The composition of DPMAS plus PE therapy was the same as that previously described (Fig. 7)^15,16^. All patients received DPMAS therapy for 2 h, followed immediately by PE therapy with half the total plasma volume (approximately 1,500 mL) for approximately 1 h. As shown in Fig. 7, the parameters of the CRRT machine were set to a blood flow of 130 mL/min, a plasma separation flow of 1,500 mL/h, and a plasma return flow of 1,500 mL/h. Patients received 4% citrate serum sodium anticoagulation with an initial speed of 120 mL/h in the arterial segment during DPMAS plus PE therapy and received 10% calcium gluconate supplement in the venous segment with a speed of 13 mL/h during DPMAS therapy and 74 mL/h during PE therapy.

### Definition

Under normal conditions, citrate’s half-life is approximately 5 min and citrate is metabolized completely within 30 min of discontinuing a citrate infusion^31,32^. Here, we defined LDCA as the presence of citrate accumulation 2 h after RCA-ALSS we defined longer duration of citrate accumulation (LDCA) as the presence of citrate accumulation 2 h after RCA-ALSS therapy concluded. Citrate accumulation was defined as the value of the ratio of Ca_tot_ to Ca_ion_ greater than or equal to 2.5 (Ca_tot_/Ca_ion_ ≥ 2.5)^8,33^.

### Statistical analysis

Patients were randomly divided into a derivation cohort and a validation cohort using SPSS software (IBM SPSS) with a ratio of 2:1. The *t* test and the *U* test were performed for quantitative data of normal distribution and that of non-normal distribution, respectively. The chi-squared test or Fisher’s exact test was performed to calculate differences between qualitative data. The predictors for LDCA in the derivation cohort were analyzed by logistic regression in univariate analysis. For any variables with *p* ≤ 0.1 in the univariate analysis, the backward stepwise (likelihood ratio) method was performed in a multivariate analysis. The predictors obtained from the derivation cohort were then tested in the validation cohort. The predictive model was also tested in the validation cohort. Predictive factors with an AUROC > 0.750 in the derivation cohort that was equivalent or greater in the validation cohort was used to derive our predictive R-CA model. With the purpose of deriving a simple, specific predictive score for patients treated with RCA-ALSS therapy, we included clinically relevant characteristics and laboratory parameters observed at baseline. An ordinal grading (0–2) was performed for individual parameters by comprehensively considering their cut-off values of AUROCs predicting the probability of LDCA, ACLF diagnostic criteria^19,20^, and clinically significant values. A score was obtained by combining the individual grade of all the significant parameters. Multiple comparisons of the score with other predictors were performed by AUROC. Statistical significance was set at *p* < 0.05. The statistical tests were performed using SPSS v.24 (IBM SPSS), except multiple comparisons of AUROCs, which were performed using MedCacl v.19 (MedCalc Software). The figures of AUROCs and linear regression lines were drawn using MedCacl v.19 (MedCalc Software) and GraphPad Prism 6 (GraphPad Software Inc.), respectively.

## Data Availability

The datasets used and/or analyzed during the current study are available from the corresponding author on reasonable request.
